# Maladie de Verneuil: un diagnostic à ne pas méconnaitre devant une lésion tumorale vulvaire et périnéale

**DOI:** 10.11604/pamj.2018.31.76.17107

**Published:** 2018-10-03

**Authors:** Anis Haddad, Olfa Zoukar, Sonia Hammami

**Affiliations:** 1 Service de Gynécologie Obstétrique, Centre Hospitalo-Universitaire Fattouma Bourguiba de Monastir, Rue du 1^er^ Juin 1955, Monastir 5000, Tunisie; 2 Services d’Endocrinologie et de Médecine Interne, Centre Hospitalo-Universitaire Fattouma Bourguiba de Monastir, Rue du 1^er^ Juin 1955, Monastir 5000, Tunisie

**Keywords:** Hidradenite suppurative, cancer vulve, diagnostic clinique, complications, traitement, Suppurative hidradenitis, vulvar cancer, clinical diagnosis, complications, treatment

## Abstract

Les auteurs rapportent le cas d'une patiente de 58 ans, nulligeste, aux antécédents de maladie de Niemann-Pick qui a consulté pour une tuméfaction vulvo-périnéale de 10 cm, fluctuante et fistulisé avec une ulcération. La patiente était fébrile et présentant un état général altéré. Cette masse a fait craindre le développement d'une tumeur maligne vulvaire nécrosée et infectée sur une dermatose chronique. Elle a eu une incision en vue de drainage des collections purulentes associée à des biopsies multiples qui ont infirmé la présence d'un processus néoplasique. L'association lésionnelle et leurs distributions ont fait porter alors le diagnostic d'une hidradenite suppurative ou maladie de Verneuil. Ensuite le problème posé était celui du choix de la conduite de prise en charge ultérieure parmi les traitements médicamenteux et chirurgicaux décrits. Les auteurs discuteront à travers ce cas et une brève revue de la littérature récente les modalités du diagnostic, les complications et la prise en charge thérapeutique de cette maladie.

## Abstract (en)

We here report the case of a 58-year old nulliparous patient with a history of Niemann-Pick disease who presented with vulvoperineal, fluctuant, fistulized swelling measuring 10 cm associated with ulceration. The patient was febrile with impaired general state This mass caused some concern about necrotic and infected malignant tumor of the vulva associated with chronic dermatosis. Incision and purulent drainage associated with multiple biopsies excluded the presence of a neoplastic process. The diagnosis of suppurative hidradenitis or Verneuil’s disease was based on the associations between lesions and their distributions. Then, the problem that arose was that of the choice between drug treatment and surgical procedures. This study, including a short review of the recent literature, aims to outline the diagnostic modalities, the complications and the therapeutic management of this disease.

## Introduction

La maladie de Verneuil, aussi appelée hidrosadénite suppurative, est une inflammation nodulaire chronique et suppurante de la peau qui touche préférentiellement les zones du corps où il existe des glandes de la sueur de type apocrines. Ces glandes sont présentes au niveau de la peau des régions ano-périnéales, des plis de l´aine, des aisselles et les mamelons chez la femme. Elle reste longtemps méconnue, vue son installation insidieuse sur de longues années. Le diagnostic peut être porté hâtivement pour une autre dermatose aux stades de début [1]. C'est une affection d'évolution chronique par poussées occasionnant tuméfactions inflammatoires douloureuses et fistules suintantes du pus malodorant. Elle constitue lit de développement de cancers épidermoïdes des régions vulvaires, périnéales et vulvaires [2]. Par conséquent sa prise en charge est au long cours qui pose plusieurs problèmes allant du diagnostic positif et de la conduite thérapeutique approprié à la surveillance attentive et régulière pour ne pas ignorer une dégénérescence maligne. Les auteurs proposent, à travers un cas et une revue de la littérature, d'étudier ces différents problèmes posés par cette pathologie.

## Patient et observation

**Figure 1:**
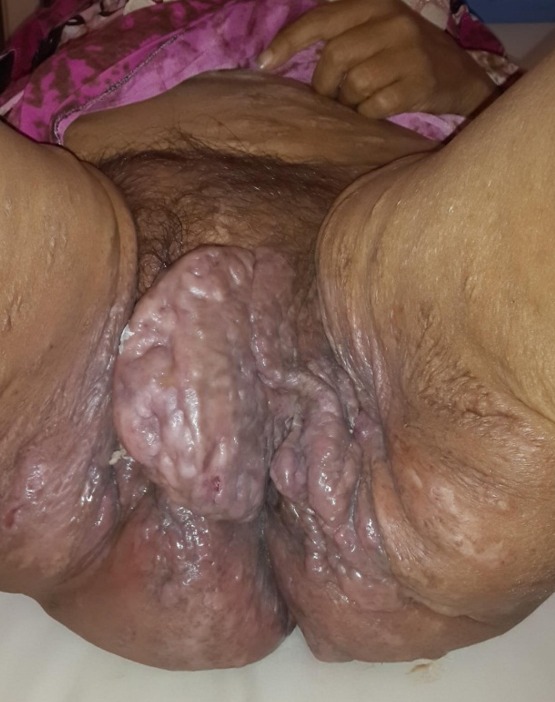
Masses nodulaires vulvo-périnéales

**Figure 2:**
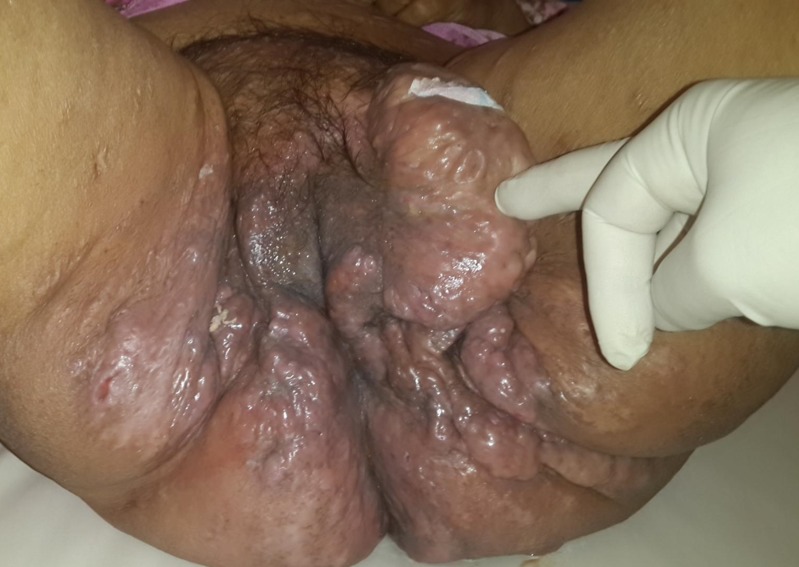
Volumineuse masse vulvaire gauche

**Figure 3:**
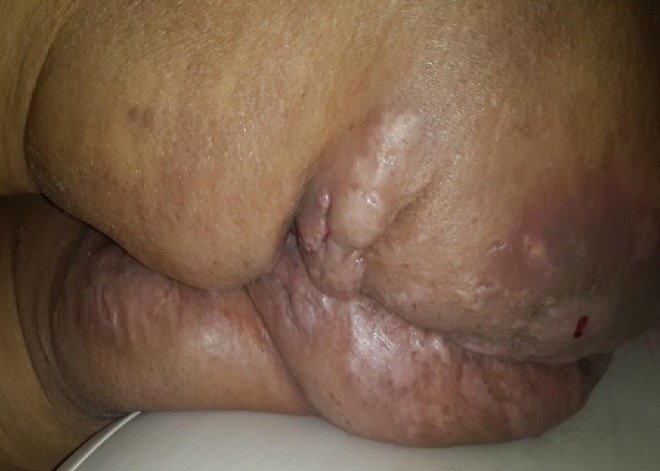
Lésions des plis fessiers et des faces internes des cuisses

Il s'agit d'une dame nulligeste de 58 ans, ménopausée depuis 10 ans. Elle était irrégulièment suivie au service de médecine interne pour une maladie Niemann Pick. Elle nous a été adressée pour lésions cutanés vulvo-périnéales pseudos tumorales multiples dont la plus volumineuse de 10 cm prend toute la grande lèvre gauche, ulcérée et faisant suspecter un cancer de la vulve infecté. Ces lésions prenaient toute la région vulvaire et s'étendant latéralement aux plis inguinaux et aux faces internes des cuisses (Figure 1, Figure 2). Elles s'étendaient également au périnée et aux fesses notamment les zones entourant les plis (Figure 3). En fait il s'agissait de lésions nodulaires variables en taille et en consistance. La peau est par place induré ou œdématié et épaisse. Les plus grosses lésions vulvaires sont fluctuantes à la palpation, sièges de nombreuses fistules et faisant sourdre un liquide séreux purulent à la pression. Ailleurs la peau est le siège de cicatrices rétractiles. Ces lésions sont invalidantes pour la patiente dont l'état générale était altéré avec fièvre, amaigrissement et difficulté à la marche. La patiente a eu une incision de la grossesse lésion. Celle-ci a assuré son drainage en effondrant de multiples logettes de pus associé à des prélèvements biopsiques. Ces derniers ont montré une atteinte inflammatoire prononcée sans signes de malignité. Il s'agissait d'un infiltrat inflammatoire polymorphe localisé surtout autour des glandes apocrines dont la lumière est dilatée associé à de la nécrose et une hyperkératose. Ainsi le diagnostic d'une maladie de Verneuil ou hidradenite suppurative a été porté surtout devant l'aspect clinique des lésions, leurs distributions et leurs associations à d'autres lésions d'âges différents extra périnéales. La prise en charge ultérieure a consisté en une antibiothérapie à base d'amoxicilline-acide clavulanique et des quinolones associés aux soins locaux et des anti-inflammatoires. Cette conduite a permis d'améliorer l'état suppuratif et douloureux de la patiente sans plus. Après un entretien a été fait entre la patiente et le chirurgien plasticien sur les modalités d'une chirurgie d'exérèse étendue, ses avantages et ses risques. La décision était alors de se contenter d'un traitement médical et d'une surveillance régulière.

## Discussion

La maladie de Verneuil ou hidradenite suppurative (HS) est une affection cutanée inflammatoire suppurative et douloureuse évoluant par poussées entrainant fistules et sclérose. Elle touche la peau dotée de glandes sudoripares apocrines principalement les régions ano-vulvo-périnéales, les plis inguinaux, inter fessiers, axillaires et sous mammaires [1]. La prévalence de cette affection varie de 0,05 % à 4,1 % selon les auteurs [2, 3]. Le sexe ratio est de 4 femmes / 1 homme [4]. L'étiopathogénie de la maladie reste mal élucidée mais il semble qu'elle soit multifactorielle impliquant des facteurs hormonaux, génétiques et immunologiques. Il semble que la HS correspond à une anomalie de l'épithélium cutané folliculaire, résultant d'un stress mécanique à répétition chez des personnes génétiquement prédisposés [5]. L'unité pilo-sébacée ainsi bouchée s'enflamme alors et forme un abcès. La rupture folliculaire subséquente favorise la formation de tractus sinusaux, lesquels s'infectent aussi à leurs tours. L'obésité et le tabac seraient des facteurs aggravants [5]. Quant à l'infection bactérienne, elle est la conséquence du processus inflammatoire et non pas l'inverse [6]. Une association à d'autres maladies auto-immunes a été signalée pour la maladie du Crohn et la spondylarthrite ankylosante [7]. A notre connaissance nous rapportons le premier cas associé à la maladie de Niemann-Pick. S'agit-il d'une association fortuite? Le diagnostic de la HS est avant tout clinique qui reste difficile à poser au stade de début. Il est insidieux et commence par une gêne légère non spécifique à type d'érythème, de brulure et de prurit. Si bien que 12 ans ou plus peuvent s'écouler entre l'apparition des symptômes et le diagnostic [8]. La HS évolue, par la suite, pour former des nodules profonds sensibles au toucher, qui s'élargissent, confluent et se transforment en abcès douloureux [5]. Ces derniers vont se fistuliser laissant soudre du pus malodorant. Cette lésion peut persister donnant lieu à d'autres trajets fistuleux ou cicatriser de façon rétractile dite en « patte de crabe ». Le diagnostic différentiel peut se discuter avec : érysipèle, furonculose, Kyste épidermoïde, la lymphogranulomatose-vénérienne et la tuberculose. À un stade plus tardif, les lésions se multiplient, des lésions jeunes coexistant avec des lésions suppurées plus évoluées et des cicatrices. La maladie évolue par poussées successives et imprévisibles. Il existe des formes bénignes limitées mais aussi des formes très extensives réellement handicapantes. La sévérité des lésions peut être évaluée selon la classification de Harley ou mieux par le score objectif de Sartorius [9]. Notre patiente avait un stade avancé de la maladie. Le diagnostic de la HS repose sur la constatation de lésions typiques siégeant dans des zones typiques et évoluant de façons chroniques par poussés. La HS peut se compliquer d'infections graves à type d'érysipèle, de fasciite nécrosante et de septicémie. Elle peut être responsable de fistules urétrales, vésicales, vaginales et rectales. Les cicatrices rétractiles et les brides gênent la mobilité et l'activité sexuelle. Par conséquent le retentissement psychologique et l'altération de la qualité de vie sont importants [10]. Quoiqu'il en soit la complication la plus redoutable et qu'on doit la guetter notamment après une longue évolution de la maladie reste le développement sur cette inflammation chronique d'un cancer vulvaire, périnéale ou anale.

Dans une revue systématique récente de la littérature, Makris GM et coll [11] ont trouvé qu'il s'agissait de cancers épidermoïdes survenant après une évolution de plus 15 ans de la HS et sont plutôt de mauvais pronostics et découverts déjà à un stade métastatique. Maclean GM et Coleman DJ [12] vont jusqu'à considérer la HS comme lésion pré néoplasique et de ce fait doit être traitée de manière agressive par une chirurgie d'exérèse large. Dans notre cas la grandeur de la tuméfaction vulvaire gauche associée à des fistules et une ulcération ont fait suspecter fortement la dégénérescence de la maladie. La patiente a eu alors un drainage chirurgical associé à des prélèvements biopsiques multiples infirmant la cancérisation. Le traitement repose sur des moyens médicamenteux et chirurgicaux. La prise en charge médicale est recommandée dans les stades précoces, alors que la chirurgie doit être pratiquée après la formation d´abcès, de fistules, de cicatrices et de sinus [5]. Le traitement médical fait appel dans tous les cas à des mesures d'hygiène tel que l'arrêt du tabac, la lutte contre l'obésité, le port de vêtements non serrés et l'utilisation de savons antiseptiques. Ailleurs il fait appel aux antibiotiques, aux rétinoïdes, à l'anti-androgène aux immunosuppresseurs et aux biothérapies [2]. Les antibiotiques peuvent soulager une poussée mains ne guérissent pas la suppuration. Ils doivent êtres alors obligatoirement prolongés jusqu'à 6 mois. Il est recommandé d'associer de la rifampicine à la clindamycine ou à la moxifloxacine [2]. Notre patiente avait bénéficié après le geste chirurgical de drainage d'une antibiothérapie prolongée à base d'amoxicilline-acide clavulanique et d'ofloxacine ce qui a permis de la soulager du point de vue douleurs et inflammation. Les anti-TNF (Infliximab, adalimumab) sont efficaces mais leur coût et les risques liés à l'immunosuppression les réservent aux échecs des autres traitements [2]. Le traitement chirurgical est d'abord celui des abcès douloureux. Il n'a pas de spécificité et associe une incision, un méchage et une cicatrisation dirigée. L'exérèse du territoire apocrine lésé est le seul traitement laissant espérer une guérison. Les exérèses partielles des territoires apocrines exposent aux récidives. L'exérèse large assure moins de risque de récidive mais expose à une morbidité plus importante comme l'infection, le saignement et les cicatrices rétractiles [13]. Elle est nécessaire quand la HS est compliquée d'un cancer ou d'une amylose [2]. Pour notre patiente, vue l'étendue des lésions et l'altération de son état générale, nous avons opté après avoir éliminé un cancer de la vulve pour un traitement médical prolongé ce qui a amélioré son état sans la faire guérir de son HS.

## Conclusion

La maladie de Verneuil est une maladie cutanée nodulaire chronique et suppurative d'expression clinique polymorphe. Le diagnostic repose sur des lésions typiques ayant une distribution typique et une évolution chronique par poussés. Au niveau périnéal et vulvaire elle peut faire évoquer une pathologie maligne surtout qu'elle constitue un facteur de risque. Sa prise en charge est multidisciplinaire médicale et chirurgicale. La surveillance doit être régulière et doit guetter la dégénérescence de la maladie.

## Conflits d’intérêts

Les auteurs ne déclarent aucun conflit d'intérêts.
